# First‐time characterization of viable but non‐culturable *Proteus mirabilis*: Induction and resuscitation

**DOI:** 10.1111/jcmm.15031

**Published:** 2020-02-07

**Authors:** Reham Wasfi, Ghada Refaat Abdellatif, Hisham Mohamed Elshishtawy, Hossam M. Ashour

**Affiliations:** ^1^ Department of Microbiology and Immunology Faculty of Pharmacy October University for Modern Sciences and Arts (MSA) Giza Egypt; ^2^ Department of Microbiology and Immunology Faculty of Pharmacy Ahram Canadian University (ACU) Giza Egypt; ^3^ Microbial Genetics Laboratory Agricultural Genetic Engineering Research Institute (AGERI) Agricultural Research Center (ARC) Giza Egypt; ^4^ Department of Biological Sciences College of Arts and Sciences University of South Florida St. Petersburg St. Petersburg Florida; ^5^ Department of Microbiology and Immunology Faculty of Pharmacy Cairo University Cairo Egypt

**Keywords:** *Proteus mirabilis*, resuscitation, VBNC

## Abstract

Pathogenic bacteria can enter into a viable but non‐culturable (VBNC) state under unfavourable conditions. *Proteus mirabilis* is responsible for dire clinical consequences including septicaemia, urinary tract infections and pneumonia, but is not a species previously known to enter VBNC state. We suggested that stress‐induced *P. mirabilis* can enter a VBNC state in which it retains virulence. *P. mirabilis* isolates were incubated in extreme osmotic pressure, starvation, low temperature and low pH to induce a VBNC state. Resuscitation was induced by temperature upshift and inoculation in tryptone soy broth with Tween 20 and brain heart infusion broth. Cellular ultrastructure and gene expression were examined using transmission electron microscopy (TEM) and quantitative real‐time polymerase chain reaction (qPCR), respectively. High osmotic pressure and low acidity caused rapid entry into VBNC state. Temperature upshift caused the highest percentage of resuscitation (93%) under different induction conditions. In the VBNC state, cells showed aberrant and dwarf morphology, virulence genes and stress response genes (*envZ* and *rpoS*) were expressed (levels varied depending on strain and inducing factors). This is the first‐time characterization of VBNC *P. mirabilis*. The ability of *P. mirabilis* pathogenic strains to enter a stress‐induced VBNC state can be a serious public health threat.

## 1 INTRODUCTION

Under stressful environmental conditions, some bacteria were reported to enter a phase called the viable but non‐culturable state (VBNC).^1^ Bacteria in the VBNC state are characterized by low metabolic activities and loss of culturability.^2^ Cells in the VBNC state are still considered viable because they retain some properties, including metabolic activity, membrane potential, membrane integrity, intracellular enzymatic activity, gene expression and retention of plasmids.^3^ VBNC‐inducing stresses include nutrient starvation,^4^ incubation outside normal temperature,^5^ high osmotic pressure,^4^ sharp pH changes,^4^ antibiotic pressure 6 and heavy metals.^7^ Resuscitation of VBNC cell can occur upon removal of the inducers,^8^ exposure to amino acids, using host cells such as embryonated chicken eggs and exposure to resuscitation promotion factor.^9^ Pathogenic bacteria that enter the stress‐induced VBNC phase upon release from the human body to the environment can run undetectable or underestimated and can thus threaten public health.^10^

Some bacteria in the VBNC state were found to display manifestations of their virulence attributes while others can lose part of their virulence properties.^11^ Bacteria can also display virulence traits upon resuscitation.^12^ Furthermore, cells in the VBNC state were found to show additional virulence traits, such as antibiotic resistance which were not shown in their normal state.^13^ This might explain recurrent infections by antibiotic‐resistant bacteria that enter VBNC state and then retain their full activity upon condition improvement.^7^ A number of pathogenic bacteria have been reported to enter the VBNC state including *Campylobacter* spp., *Escherichia coli*, *Francisella tularensis, Helicobacter pylori, Legionella pneumophila*, *Listeria monocytogenes*, *Mycobacterium tuberculosis*, *Pseudomonas aeruginosa*, several *Salmonella* and *Shigella* sp., and numerous pathogenic *Vibrio* sp.^14^ However, up to our knowledge and by searching the PubMed database (https://www.ncbi.nlm.nih.gov/pubmed/), there were no studies related to the VBNC state in *P. mirabilis*.

*Proteus mirabilis* bacteria are Gram‐negative, facultative anaerobes that can be responsible for dire clinical consequences including septicaemia, urinary tract infections and pneumonia.^15^ *Proteus *sp. possess several virulence factors including fimbriae, flagella, urease enzyme, lipopolysaccharide, capsular polysaccharide and metalloproteinase (serralysin).^16^ *Proteus *sp. are normal flora in the intestinal tract.^17^ Their presence in soil or water is an indicator of faecal pollution and can consequently cause infection to human consuming contaminated water or contaminated seafood.^18^ Some sea animals are reported to absorb and accumulate *Proteus* spp. such as sponges (*Spongia offinialis*),^19^ oysters 20 and turtles.^21^ *Proteus* sp. is the causative agent for many urinary tract and wound infections as well as nosocomial infections.^22^ Studies have reported similarities between *Proteus* sp. isolated from human intestine and those causing urinary tract infection (autoinfection) 23 or food poisoning.^21^

We suggested that *P. mirabilis* can enter a VBNC state and that this VBNC state impacts gene expression and cellular ultrastructure of the VBNC cells as compared to control cells. We also studied different *P. mirabilis* VBNC‐inducing factors and resuscitating conditions. To the best of our knowledge, this is the first‐time characterization of VBNC *P. mirabilis*.

## 2 MATERIALS AND METHODS

### 2.1 Bacterial strains

Three *P. mirabilis* clinical isolates were recovered from patients in Kasr Al‐Aini Hospital, Cairo, Egypt. Approvals from the research ethics committees of the hospital and the faculty of Pharmacy, October University for Modern Sciences and Arts, Giza, Egypt were obtained prior to conducting the study. Two isolates, P6 and P7, were recovered from urinary tract infections, and the isolate, coded P3, was recovered from wound infection. Written consents were obtained from all participants in the study.

### 2.2 Induction of the VBNC state

The VBNC state induction was done according to Pinto et al 24 with some modifications. *P. mirabilis* strains were grown overnight in tryptone soy broth (TSB) at 37°C with shaking. The culture was centrifuged at 5000 rpm for 10 minutes at 5°C, and pellets were washed twice by sterile saline solution. The resulting pellets were resuspended in sterile deionized water, and the cell concentration was adjusted to be equivalent to McFarland 0.5 (1.5 × 10^8^ CFU/mL). A volume of 400 µL was used to inoculate 40 mL of the induction medium, in a 50‐mL falcon tube, to reach a final density of approximately 10^6^ CFU/mL. Different induction media were used to create different stressful conditions for isolates: (a) deionized water, for starvation and low osmotic pressure stress; (b) deionized water + NaCl (0.9%), for starvation stress; (c) deionized water + NaCl (0.9%) at pH 5, for starvation and acidic condition stresses; (d) deionized water + NaCl (4%), for starvation and high osmotic pressure stresses, and (e) deionized water + NaCl (7%), for starvation and high osmotic pressure. Each induction condition was done in duplicate, and tubes were incubated at 4°C, without shaking and in dark. Culturability on TSA was assessed every week in duplicate plates, and time required for loss of culturability was recorded. Test of culturability was done by testing the growth of 1 mL of culture, and then, when no growth is detected, 10 mL of culture was centrifuged and resuspended in 1 mL and repeated subculture was done. Heat killed bacteria at 100°C for 15 minutes were used as control cells.

### 2.3 RNA isolation and cDNA preparation

30 mL of each VBNC sample was centrifuged at 10 000 g for 10 minutes at 4°C. Total RNA was extracted from cultures using the SV Total RNA Isolation^®^ System (Promega). Extraction was done according to the manufacturer's instructions. RNA was stored in RNase‐free water and then stored at −80°C until use. RNA quantity and quality were measured at wavelengths 260 and 280 nm using ND‐1000 spectrophotometer (NanoDrop Technology). RNA quality was also tested by agarose gel electrophoresis. The RNA samples were treated with RNase‐free DNase I (New England Biolab). Samples were confirmed to be free of genomic DNA by 40 cycles of conventional PCR using 16S rRNA primers (long amplicon, ~1.5 kb). After confirming the absence of genomic DNA by agarose gel analysis, ~l µg DNase‐treated RNA was subjected to reverse transcription using the SensiFastTM cDNA synthesis kit (Bioline) following the supplier's directions (Table 1). Control bacteria for qRT‐PCR were prepared by inoculation in BHI medium and incubation at 37°C for the late exponential phase. This was followed by total RNA extraction using the same methodology that was implemented for the VBNC samples.

**Table 1:** List of oligonucleotide sequences and the corresponding annealing temperatures

Target gene a	Primer sequence 5’‐3’	Ta*	Amplicon size (bp)	Reference
***FtsZ***	For: AAGGCGAAGATCGTGCTGAA Rev: TGCACGAATGGTGTTACCGA	55	161	This study
***FliL***	For: TATTTACCCGAAGTTCGTAGT Rev: GGTAAACAGTACATCGGAGAG	55	150	
***ZapA***	For: TATGCGGAAGATAGTCGCCAA Rev: AGTCGTATTTGCGCCATAGAG	55	150	
***Rpos***	For: AGGCCTTATTCGTGCTGTTG Rev: ATCATCGACCGGTTTATCCA	55	200	
***EnvZ***	For: GCTTGGTTTCAAGTTGAAGAC Rev: TAGACCTAAACCCGCACC	55	120	
***LuxS***	For: TTCGCCAATGGGATGTCGTA Rev: TCCATAGCTGCCTTCCATGC	55	93	
***16s rRNA*** b *** (short amplicon)***	For: CACACTGGAACTGAGACAC Rev: CTTCTTCTGCGGGTAACG	55	189	69
***16s rRNA*** c *** (long amplicon)***	For: AGA GTT TGA TCC TGG CTC AGU Rev: GGT TAC CTT GTT ACG ACT T	47	1465	70

Note

Ta* annealing temperature.

a *ftsZ* gene, coding for cell division protein; flil, coding for flagellar basal body protein; *zapA,* serralysin *(metalloprotease); rpoS,* stress stationary phase regulator; *envZ,* two‐component sensor histidine kinase; and *luxS*, s‐ribosyl homocysteine..

b 16 s rRNA primer used in VBNC validation and in qPCR.

c 16 s rRNA primer used for confirmation of complete removal of genomic DNA.

### 2.4 Validation of the VBNC state

The RNA expression was used as tool indicating viability of cells.^25^ After loss of culturability on routine media by VBNC‐induced cells, the continuous expression of the housekeeping genes 16S rRNA (short amplicon, 189 bp) and *Rpos* was used as confirmation for cell viability under different stressful conditions using specific primers (Table 1). The amplicon was detected using gel electrophoresis for 16S *rRNA* gene and qRT‐PCR for *RpoS* gene.

### 2.5 Resuscitation of the VBNC cells

Different conditions of resuscitation were used: (a) temperature upshift, (b) inoculation in TSB with added nutrients of Tween 20 (3% v/v) and (c) inoculation in low‐nutrient medium BHI (1/8 concentration).

Resuscitation by temperature upshift was performed according to Zeng et al 26 with modifications. 10 mL of VBNC culture was placed in the incubator at 10°C, and then, the temperature was increased gradually by 5°C every 60 minutes till the temperature reaches 35°C. At this point, 2 mL out of the 10 mL VBNC suspension was seed cultured in TSA plates to check the ability of the cells to regain its culturability.

10 mL medium was inoculated with 20 µL from the VBNC suspension. The inoculated media included the following: TSB with added Tween 20 and low‐nutrient medium BHI (1/8 concentration). Inoculated media were incubated at 37°C for 72 hours, and then, the appearance of turbidity indicated resuscitation of VBNC cells and regaining of the culturability of VBNC cells.^27^

### 2.6 Quantitative PCR conditions

We studied the change in gene expression of the following genes: stress response genes (*rpoS* and *envZ*), virulence genes (flil, *zap*A and *luxS*) and genes for cell division (*ftsZ*). Expression in VBNC cells was compared to control cells. The primers were designed using the complete genome sequence of *P. mirabilis* (HI4320) obtained from the NCBI database (NCBI accession no. https://www.ncbi.nlm.nih.gov/nuccore/NC_010554). Primers for the qPCR used in the current study (Table 1) were synthesized by Invitrogen^®^ (Massachusetts, USA).

Quantitative real‐time reverse transcription polymerase chain reaction (qRT‐PCR) was performed by Applied Biosystems StepOne^™^ Instrument using SensiFast^™^ SYBR HiRox Master (Bioline, Massachusetts. USA). All reactions (20 μL) were performed using three technical replicates. Each reaction mixture contains 100 ng cDNA and 400 nmol/L primers per reaction. The RT‐PCR cycling conditions were as follows: one cycle with 95°C for 2 min; then 40 cycles of denaturation at 95°C for 5 seconds, annealing at 55°C for 10 seconds, and extension and fluorescent data collection at 72°C for 20 seconds. A dissociation curve was generated at the end of each reaction. In all qPCR runs, negative controls without template were run in parallel. The *16s rRNA* gene (housekeeping gene) was selected as the internal control based on the results of BestKeeper^®^ software tool.^28^ The relative mRNA levels of genes of interest were determined and normalized to the expression of the housekeeping gene using the ∆∆ CT value analysis.^29^ The qPCR data were expressed as the fold change in expression levels of genes in *P. mirabilis* potential VBNC as compared to their levels in the control cells unexposed to stress (calibrators) grown in BHI medium and incubated for the late exponential phase. Only genes with a relative 2^−∆∆C^ value above 1.0 or below 1.0 were considered significant. [Correction added on 17 February 2020: 2^∆∆C^ has been changed to 2^−∆∆C^ in the preceding sentence.]

### 2.7 Transmission electron microscopy (TEM)

The structures of control, VBNC and resuscitated cells were investigated using transmission electron microscope (TEM). Cells were harvested by centrifugation, and the bacterial pellets were washed three times with PBS. Cell fixation and processing steps were performed according to the method described in Henriques et al.^30^ Micrographs for each sample were captured at 10 000 g magnification power, and cells were observed for structural alterations using JEM 100 CX transmission electron microscope (JEOL, Japan) operated at 80 kV. [Correction added on 17 February 2020: 10 000× had been changed to 10 000 g in the preceding sentence.]

### 2.8 Statistics

Results of three replicates were analysed for statistical significance with PRISM 5 (GraphPad). A one‐way analysis of variance (ANOVA) was performed. Data comparisons were performed using Bonferroni's correction for multiple testing.

## 3 RESULTS

**Figure 1:**
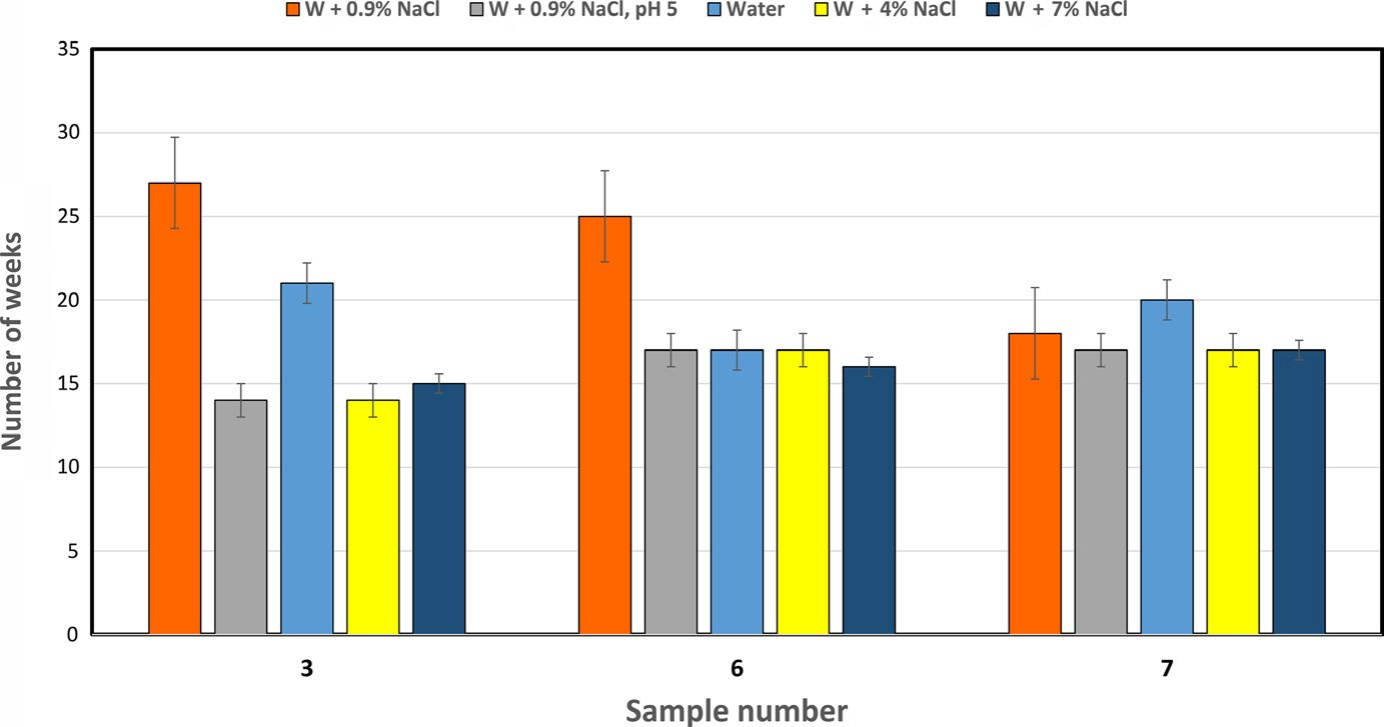
The time required for *Proteus mirabilis* cells to lose culturability under various VBNC induction condition. All samples were incubated at 4°C in water microcosm, hence exposed to cold and starvation stresses. Induction media include the following: (A) Water (W) + 0.9% NaCl. (B) Water (W) + 0.9% NaCl, pH 5. (C) Water (W). (D) Water (W) + 4% NaCl. (E) Water (W) + 7% NaCl

The samples were exposed to different VBNC induction conditions which include a combination of either acidic or osmotic pressure in addition to both cold and starvation stresses. The number of weeks taken by each of the three samples to enter in the VBNC state varied from 14 to 26 weeks according to the inducing condition (Figure 1). Isolates incubated under isotonic pressure (deionized water + 0.9% NaCl) lost their ability for culturing after a time ranging from 18 to 26 weeks (Average 22 weeks). High osmotic pressure (deionized water + NaCl 4% or 7%) and acidic conditions caused rapid entry into VBNC state compared to isotonic water microcosm. The time range required for entry of low osmotic pressure (deionized water only) was between 16 and 21 weeks (average 18.5 weeks).

**Figure 2:**
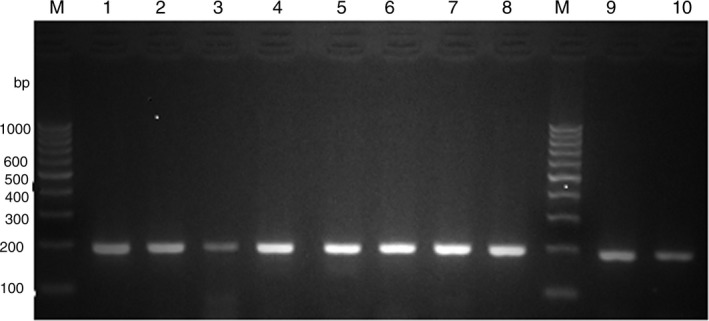
Gel electrophoresis of PCR product amplified with the 16s rRNA primer set (189 bp) of the cDNA produced by reverse transcription of RNA extracted from *P. mirabilis* samples 3 (lanes 1‐5) and 6 (lanes 6‐10) exposed to different VBNC conditions at 4°C. Media used include the following: W, deionized water (lanes 1,6); S, deionized water + NaCl 0.9% (lanes 2,7); deionized water + NaCl 0.9% at pH 5 (lanes 3,8); 4%, deionized water + NaCl 4% (lanes 4,9); deionized water + 7% NaCl (lanes 5,10). Lane Lad: represents the molecular size ladder 100‐bp DNA ladder

**Figure 3:**
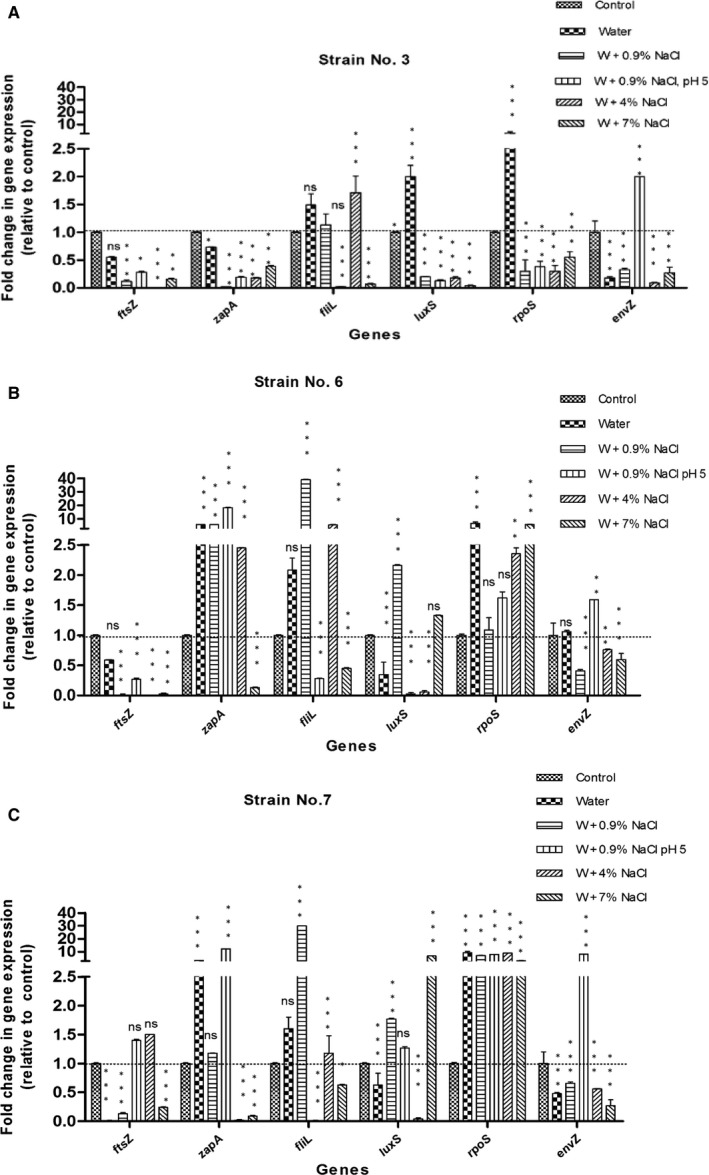
Transcription levels of selected genes (*ftsZ*, *zapA*, *fliL*, *luxS*, *rpoS* and *envZ*) of each *P. mirabilis* strain [3(A), 6 (B), 7 (C)] in the VBNC state, incubated in various environmental conditions. The conditions used include: water microcosm, water + 0.9% NaCl, water + 0.9% NaCl, pH5, water + 4% NaCl and water + 7% NaCl. Standard deviations are indicated as vertical bars. Asterisks indicate statistically significant differences in the expression of each gene between VBNC samples and control, as analysed using the one‐way ANOVA with Bonferroni's correction for multiple testing (*P ≤ .05; **P ≤ .01; ***P ≤ .001; *ns*, no significant difference)

Samples that lost their ability for culturing were confirmed to be viable by subjecting them to total RNA extraction followed by reverse transcription to cDNA. After amplification by conventional PCR, the expression of the 16S *rRNA* and *RpoS* genes was detected by gel electrophoresis (Figure 2) and qRT‐PCR (Figure 3), respectively. The continuous expression of these genes was detected in all samples under different VBNC induction conditions, and this confirmed the entry into the VBNC state. No detectable 16s *rRNA* or *Rpos* in killed control cells after 48 hours of heat treatment.

As shown in Table 2, methods of resuscitation included temperature upshift and using different media. Samples incubated in water microcosm showed the highest ability to regain culturability with different resuscitating factors. Raising the incubation temperature from 4°C to 35°C caused higher rate of successful resuscitation that reached 93% of samples incubated in starvation conditions. The low nutrient medium BHI (1/8 concentration) showed the lowest ability to resuscitate different strains with an average resuscitation percentage of 20%. On the other hand, VBNC cells induced by starvation only (water + 0.9% NaCl) were not resuscitable by BHI (1/8). TSB media with additive Tween 20 can resuscitate VBNC cells under the ‘water only’ condition (Figure 1).

**Table 2:** Resuscitation of VBNC‐induced *Proteus mirabilis* strains

Resuscitating conditions	Strains														
	3					6					7				
	W	S	A	F	V	W	S	A	F	V	W	S	A	F	V
Temp. Upshift^a^	+	+	+	−	+	+	+	+	+	+	+	+	+	+	+
TSB+ 3% Tween 20^b^	+	−	+	+	−	+	−	+	+	+	+	−	+	+	+
BHI (1/8)^c^	−	−	−	−	−	+	−	−	−	−	+	−	+	−	−

Note

(+) indicates growth, (‐) indicates no growth.

VBNC induction conditions; W = water; S = W+0.9% NaCl; A = W+0.9% NaCl, pH 5; F = W+4% NaCl; V = W+7% NaCl.

a Temperature upshift: increasing incubation temperature from 10 to 35°C at a rate of 5°C every 60 min, followed by culturing in tryptone soy agar medium (TSA).

b Twenty microlitres of VBNC‐induced culture inoculated in TSB with additive Tween 20 (3%).

c Twenty microlitres of VBNC‐induced culture inoculated in low concentration BHI broth (1/8 concentration).

We assessed the relative gene expression of tested strains after their entry into the VBNC state as compared to control bacteria grown in TSB to the late exponential phase. Genes studied included stress response genes (*rpoS, envZ*), virulence genes (*zapA*, *fliL*), autoinducers coding genes (*luxS*) and the gene coding for septum formation during division (*ftsZ*). Gene expression was normalized relative to the 16S rRNA gene. The *ftsZ* gene expression was down‐regulated in all VBNC cells (Figure 3).

The stress response gene *rpoS* was significantly up‐regulated under all conditions for isolate number 7 (≃10‐fold). Up‐regulation was observed in strain 6 under the conditions of water, NaCl 4% and NaCl 7%, by 10‐, 2‐ and 10‐fold. In strain 3, significant up‐regulation (2.9‐fold) was observed only in the presence of water, whereas down‐regulation was observed in presence of all other conditions. Significant down‐regulation for the *envZ* gene was observed under all conditions except the acidic conditions, at which the *envZ* expression was significantly up‐regulated (1.5‐ to 2‐fold). The expression of the *fliL* gene coding for basal flagellar protein varied depending on the condition inducing the VBNC state. While the acidic pH caused significant down‐regulation in *fliL* gene expression, the high osmotic pressure (4% NaCl) caused a significant up‐regulation of the *fliL* gene expression (an increase of 1.7‐fold in strains 3 and 7 and an increase of 9‐fold in strain 6). The expression of the gene *zapA* coding for metalloprotease protein was variable depending on strains and conditions inducing VBNC. Significant down‐regulation was observed under all conditions with strain 3 and in the presence of high salt concentrations (4% and 7%) in strain 7. The *zapA* gene was significantly up‐regulated in isolate 6 under all conditions. The *luxS* gene coding for autoinducer 2 (AI 2) was generally down‐regulated in all tested isolates under most conditions (Figure 3).

**Figure 4:**
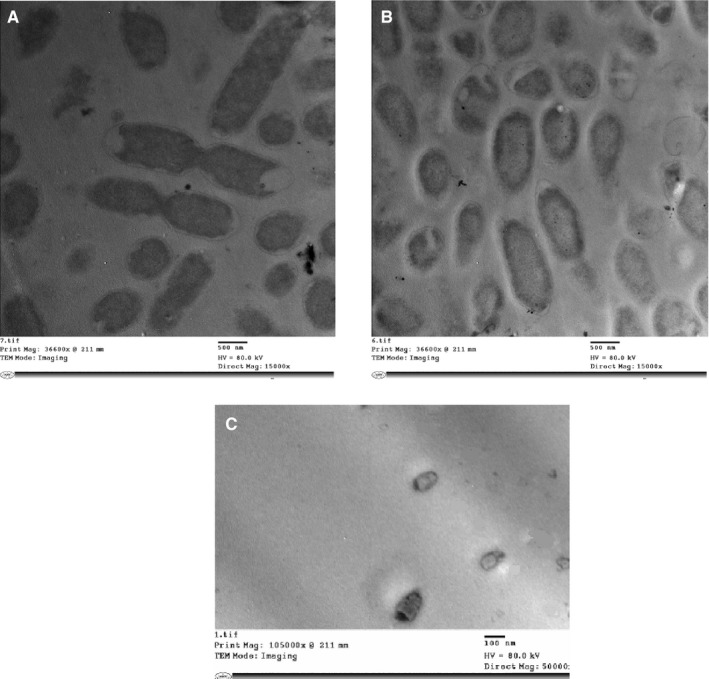
Representative TEM micrographs showing the structural changes in *P. mirabilis* (strain 6), in (A) control, (B) resuscitated and (C) VBNC state (after starvation for 182 days in water microcosm + 0.9% NaCl). Samples A and B were examined at magnification 15 000× while VBNC cells were examined at 50 000×

Strain 6 entered the VBNC state under the starvation stress (in water microcosm containing 0.9% NaCl) after 182 days. Upon TEM examination of the VBNC cells, cells were aberrant shaped, smaller in size and more rounded than control cells. They had thicker cell walls with condensed dark cytoplasm attached to the cell wall (Figure 4C). Resuscitated cells, in TSB medium containing Tween 3%, had a smaller size (Figure 4B) than actively growing cells in the late exponential phase (Figure 4A).

## 4 DISCUSSION

The VBNC state is a potential public health hazard in the environment,^7^ because bacterial cells under different environmental stresses such as low or high osmotic pressure,^31^ starvation 32 and acidic pH 33 can shift to the VBNC state which could run undetected. During the transient existence of pathogenic bacteria in the environment before infecting another person, they might be exposed to stressful conditions that cause the entry of these cells into the VBNC state.^34^ There are no previous data in the literature that characterizes *P. mirabilis* VBNC state.

In our study, the conditions used for inducing the VBNC state in *P. mirabilis* were cold temperature (4°C) and starvation accompanied by: low osmotic pressure (deionized water); high osmotic pressure (deionized water + 4 or 7% NaCl); acidic pH (deionized water + 0.9% NaCl at pH 5); and isotonic pressure (deionized water + 0.9% NaCl). The entry of *P. mirabilis* (starting count 1.5 × 10^6^ CFU/mL) into the VBNC state under cold conditions (4°C) was more rapid than at room temperature (25°C), and the maximum time required for loss of culturability was 26 weeks at 4°C and over than 65 weeks at 25°C (data not shown). The rapid entry into VBNC state at 4°C could be attributed to the inability of the cells to detoxify lethal peroxides produced under stressful conditions, because cold incubation prevents catalase activity and its de novo synthesis^35^ This can lead to an increase in toxins and failure of the antitoxin effect which is an inducer of the VBNC state.^7^

*Proteus mirabilis* showed more rapid entry (20 weeks) into the VBNC state compared to the *E coli* O157:H7 in a study 8 (34 weeks), that used similar starting bacterial concentration and similar VBNC‐inducing conditions. Thus, the speed of entry depends on the microbial species. In our study, it was noticed that exposure of *P. mirabilis* to combined stressful factors lead to more rapid entry (*P* < .01) into the VBNC state than exposure to single factor as detected in *E. coli* by Muela et al^36^ Putting the cells under starved conditions makes them more resistant to stresses such as acid and oxidizing agents than growing cells.^37^ Moreover, *Proteus* sp was found to survive in marine environments due to their halotolerance trait attributed to the polyphosphate kinase encoded by the *ppk* gene,^16^, ^38^ and ppk mutant strains of *Campylobacter jejuni* were found to exhibit decreased ability to enter the VBNC state,^39^ which could explain their ability to retain viability while losing culturability under high salt concentrations.

The entry of the *P. mirabilis* isolates into the VBNC state was confirmed by detecting the continuous expression of the 16S *rRNA* and *rpoS* genes in tested strains after losing their culturability. RNA detection was used because the half‐life of RNA is 3‐5 minutes and its continuous expression is an indication of continuous viability of bacteria that have lost their culturability.^40^ The 16S *rRNA* and *Rpos* genes were used as viability indicators as they are both housekeeping genes that need to be continuously expressed in living cells. The expression of 16S *rRNA* remains consistently high in viable cell indicating its validity as a viability indicator. Conversely, the gene is absent in dead bacterial cells after 48 hours of treatment. The *rpoS* gene is similarly absent in dead cells. This confirmatory method for entry of bacterial cells into the VBNC state was previously used in other studies.^41^, ^42^The 16s *rRNA* genes were not detected after 48 hours from heat treatment at high temperature for 15 minutes as found by McKillip  et al^43^ The *RpoS* gene was not detected in dead cells as detected by Liu et al 44

Several factors play roles in the ability of bacteria to resuscitate from the VBNC state. These factors include the particular strain used and the conditions that can induce the VBNC state and/or resuscitation^24^ . Some of the mechanisms used for resuscitation include temperature upshift and removal of environmental stressors.^8^ We have used three conditions for resuscitation including: a) temperature upshift; b) low‐nutrient medium (BHI 1/8 concentration); and c) TSB with Tween 20 (3%v/v). The most successful one was temperature upshift with a resuscitation success rate of 93.3% (14/15), which could be attributed to reaching the optimum temperature for catalase enzyme activity which can catalyse the breakdown of toxic peroxides.^35^ The lowest percentage of resuscitation was observed using BHI medium (1/8 concentration). By comparing the effect of low‐nutrient (BHI medium of 1/8 concentration) and high‐nutrient medium (TSB with additive Tween 20) on resuscitation, it was observed that the high‐nutrient medium was more efficient in resuscitating VBNC *P. mirabilis* strains than the low‐nutrient medium. This result is in contrast to what was reported by Bloomfield et al^45^ who noted that low‐nutrient environment was more efficient in resuscitation because of the toxic effect of high nutrients on cells in the VBNC state as a result of production of free radicals. Adding Tween 20 to TSB medium was assumed to reduce surface tension and improve the interaction between VBNC cells and the culture medium,^46^ in addition to acting as carbon source that supplies energy to VBNC cells.^47^

The virulence factors known to be involved in pathogenesis of *P. mirabilis* include adhesins, motility, biofilm formation, immunoavoidance, toxins and nutrient acquisition.^48^ The gene expression of six different genes in three strains (P3, P6 and P7) was studied using relative qPCR. These genes code for: septum formation (*ftsZ*), stress response regulators (*rpoS* and *envZ*), virulence traits (*zapA* and *fliL)* and autoinducer (AI‐2) production *(luxS).* On assessing the relative expression of *ftsZ* gene under different induction conditions, the expression of this gene in the tested VBNC cells was reduced by variable folds according to the stress conditions and strains. Down‐regulation of *ftsZ* expression in VBNC bacteria was previously observed.^49^, ^50^ TEMobservation of the VBNC cells of *P. mirabilis* (P6) incubated in deionized water (containing 0.9% NaCl at 4^°^Cfor 182 days) revealed that VBNC cells appear aberrant and smaller in size than normal and resuscitated cells with condensed cytoplasm at the poles of the cells, condensed chromosomal DNA and intact cytoplasmic membrane. Upon resuscitation, the bacteria increased back to the normal size. The aberrant and dwarfing shapes were also observed in the VBNC cells of *Vibrio parahaemolyticus*,^51^ *Vibrio vulnificus*,^2^ *Listeria monocytogenes*,^52^ *Yersinia pestis* 27 and *Helicobacter pylori*.^53^ Li et al 54 reported that the starvation state interferes with the normal function of penicillin binding protein (PBP) which is important for cell wall formation during cell division. This interference lead to transition in the morphology of bacteria during their conversion from viable cells to the VBNC state, from rod shaped to coccoid form. The dwarfing size is the result of catabolism of cellular components under conditions of nutrient limitation, which leads to an increase in the surface area for nutrient transportation.^55^

The entry of bacterial cells into the VBNC state provides them with necessary resistance to unfavourable stress conditions.^56^ Thus, we have studied the differential expression of the stress response genes (*rpoS* and *envZ*) under these conditions to determine its role in stress resistance. The *envZ* gene controls the outer membrane protein (Omp), and thus, it has a role in controlling membrane porins and sensing the change in environmental stimuli. Some studies suggested that *envZ* gene is vital for entry into the VBNC state and that any mutations in this gene could inhibit the entry into this state but does not affect the survival under the VBNC‐inducing conditions.^57^ In our study, the *envZ* gene, which is part of osmoregulating system *EnvZ‐OmpR*, was down‐regulated under all tested condition except in samples incubated in acidic deionized water where it is up‐regulated (1.6 to 8 folds) compared to normal cells. These results indicate that *envZ* gene has no role in cell survival under VBNC state except under acidic conditions and this observation was also noticed in a study carried by Kaeriyama et al 58 on *E coli,* where *envZ* controlled porins were up‐regulated under low osmotic pressure (0.5% NaCl) at acidic pH. Stress δ^s^ factor, coded by *rpoS* gene, controls the production of trehalose, which acts as a protective osmolyte, and is associated with persistence in the VBNC state.^59^ Mutation in this gene causes fast entry and less survival time in the VBNC state,^59^ in addition to delayed ability for resuscitation.^9^ Regulation and control of *rpoS* gene expression occurs at two levels: transcriptional and translational. There are differences between factors inducing *rpoS* gene transcription and those inducing *rpoS* mRNA translation into δ^s^ factor. *RpoS* gene transcription into *rpoS* mRNA is induced by reduced growth rate and is negatively controlled by cAMP‐CRP,^60^ while its translation into functional δ^s^ factor is induced by osmotic pressure and high cell density.^61^ In our study, the expression of *rpoS* gene was variable under combined effects of starvation with high osmotic pressure and acidic conditions. Gene expression was down‐regulated in strain 3 compared to normal cells while up‐regulation occured in strains 6 and 7. VBNC‐induced cells incubated in water microcosm showed up‐regulation in *rpoS* expression. The down‐regulation in *rpoS* gene in strain 3 might explain the lower percentage (44%) of resuscitation in this strain under different resuscitating conditions compared to strains 6 and 7, in which percentages of resuscitation were 64 and 67%, respectively.

The ability of pathogenic bacteria to retain their virulence factors in the VBNC state is strain‐dependent.^3^, ^62^ We have studied the relative expression of genes coding for selected virulence traits including flagellar basal protein (*fliL*) and metalloprotease (*zapA*). Bacterial motility increases under high osmotic pressures 63 which is retained under VBNC conditions as was observed in our study and in the study of Asakura et al^31^ on VBNC *V. vulnificus*. Conversely, the expression of *fliL* gene was down‐regulated in all tested strains under acidic pH, which lead to reduction in flagella formation and motility as reported by Soutourina et al^64^

*Proteus mirabilis* encodes a metalloproteinase, serralysin (ZapA), which was shown to cleave secretory immunoglobulins and antimicrobial peptides and hence establishing urinary tract infections.^65^ Although *zapA* is expressed at low levels in vivo, a *P. mirabilis zapA* mutant is less efficient than wild type at colonizing the urine, bladder and kidneys in murine infections.^66^ In our study, the expression of zapA gene was variable depending on the strain and the VBNC induction conditions but it persisted under unfavourable conditions indicating that *P. mirabilis* retained its metalloproteinase virulence traits during the VBNC state.

*Proteus mirabilis* possesses a *luxS* homologue and produces AI‐2, which can mediate interactions between species.^67^ Continuous expression of *luxS* gene was observed in all tested strains under VBNC conditions. However, AI‐2 does not likely contribute to pathogenicity,^67^ in spite of studies that suggest the involvement of *luxS* expression in biofilm formation.^68^ The down‐regulation of the *luxS* gene under different VBNC‐inducing conditions in our study might indicate a reduced ability of VBNC cells for biofilm formation.

In conclusion, *P. mirabilis* can enter the VBNC state under several conditions of starvation, extreme osmotic pressure, acidity and low temperature. The time required for entry into a VBNC state is dependent on the inducing condition and the bacterial strain. Removal of the cold stress by temperature upshift on VBNC cells lead to successful bacterial resuscitation. Bacterial cells under the VBNC state showed aberrant dwarf morphology and returned back to their normal shapes after resuscitation but with relatively smaller sizes as compared to normal cells. Gene expression was variable under different conditions and the continuous expression of virulence trait‐coding gene indicated that bacterial cells can retain some of their virulence properties under the VBNC state.

## CONFLICT OF INTEREST

The authors declare no competing financial interests.

## AUTHOR CONTRIBUTIONS

Dr Reham Wasfi, Dr. Ghada R. Abdellatif, Dr. Hisham M. Elshishtawy and Dr. Hossam M. Ashour contributed to the design of the study, performance of experiments, analysis of the results and writing of the manuscript.

## Data Availability

The data used to support the findings of this study is available from the corresponding author upon reasonable request.
